# Aging effects on contrast sensitivity in visual pathways: A pilot study on flicker adaptation

**DOI:** 10.1371/journal.pone.0261927

**Published:** 2021-12-31

**Authors:** Xiaohua Zhuang, Tam Tran, Doris Jin, Riya Philip, Chaorong Wu

**Affiliations:** 1 Illinois College of Optometry, Chicago, IL, United States of America; 2 Study Design & Biostatistics Center, Division of Epidemiology, University of Utah, Salt Lake City, UT, United States of America; The University of Queensland, AUSTRALIA

## Abstract

Contrast sensitivity is reduced in older adults and is often measured at an overall perceptual level. Recent human psychophysical studies have provided paradigms to measure contrast sensitivity independently in the magnocellular (MC) and parvocellular (PC) visual pathways and have reported desensitization in the MC pathway after flicker adaptation. The current study investigates the influence of aging on contrast sensitivity and on the desensitization effect in the two visual pathways. The steady- and pulsed-pedestal paradigms were used to measure contrast sensitivity under two adaptation conditions in 45 observers. In the non-flicker adaptation condition, observers adapted to a pedestal array of four 1°×1° squares presented with a steady luminance; in the flicker adaptation condition, observers adapted to a square-wave modulated luminance flicker of 7.5 Hz and 50% contrast. Results showed significant age-related contrast sensitivity reductions in the MC and PC pathways, with a significantly larger decrease of contrast sensitivity for individuals older than 50 years of age in the MC pathway but not in the PC pathway. These results are consistent with the hypothesis that sensitivity reduction observed at the overall perceptual level likely comes from both the MC and PC visual pathways, with a more dramatic reduction resulting from the MC pathway for adults >50 years of age. In addition, a similar desensitization effect from flicker adaptation was observed in the MC pathway for all ages, which suggests that aging may not affect the process of visual adaptation to rapid luminance flicker.

## Introduction

Contrast sensitivity is one of the most fundamental functions of the human visual system. It is the ability to detect spatial contrast (e.g., achromatic luminance difference between areas). Many activities of our daily life, such as finding an object, seeing stairs, and noticing a moving object, rely on this ability. Research has shown that evaluation of spatial contrast sensitivity correlates with many higher-order visual functions [1–3], provides more information about the visual system, and is a better predictor of daily visual performance than visual acuity [4, 5]. Clinically, contrast sensitivity deficits have been observed in many ophthalmic conditions, such as amblyopia [6–9], glaucoma [10–12], diabetic retinopathy [13, 14].

Similar to many other visual functions, contrast sensitivity is unavoidably affected by aging [4, 15]. Despite some discrepancies, studies have shown that contrast sensitivity deterioration starts at high spatial frequencies for individuals between 40–50 years of age, and then extends to all spatial frequencies at later ages [4, 16–18]. For instance, Derefeldt et al. (1979) showed that contrast sensitivity did not vary with age below 40 years old, whereas adults over age 60 years demonstrated significant loss of contrast sensitivity for middle and high spatial frequencies; Owsley et al. (1983) on the other hand showed a decrease in contrast sensitivity starting at 40 to 50 years of age for high spatial frequencies but not for low spatial frequencies. Nameda et al. (1989) found deterioration across all spatial frequencies at the age of 50–60 years. In addition to the age range at which deterioration starts, another interesting question about aging is, at which stage(s) a significant change occurs. A common agreement is that optical factors likely play important contributing roles in the impairment [4, 15, 19], however, the roles of neural factors remain controversial [20, 21].

The majority of the research studies and clinical assessments measure contrast sensitivity at an overall perceptual level. However, physiological and recent psychophysical studies have shown that the different visual pathways have very distinct contrast processing characteristics [22, 23], in particular, the two primary visual pathways in the primate visual system, the magnocelluar (MC) and parvocellular (PC) visual pathways. Both pathways originate at the retina and remain separate up to the primary visual cortex (V1) [24, 25]. Neurons in these two pathways (i.e., parasol and midget ganglion cells or M and P cells in the lateral geniculate nucleus [LGN]) show different response characteristics to achromatic contrast. MC cells show high sensitivity to very low contrast, and their response rate increases quickly with increasing contrast, but quickly saturates at relatively low contrast. On the other hand, PC cells show lower sensitivity to low contrast and relatively linear contrast response rate to different contrast levels [26, 27]. To fully understand the roles of neural factors in age-related deterioration, it is essential to understand how aging affects the contrast processing in each of these two pathways.

The psychophysical paradigms developed by Pokorny and Smith (1997) demonstrate the ability to measure contrast sensitivity in the MC and PC visual pathways independently. Using these paradigms, two prior studies showed aging effects on contrast sensitivity in both visual pathways [28, 29]. Contrast sensitivity in both pathways in older observers was lower than the contrast sensitivity in younger observers. However, in both studies, an older group with a mean age >70 years was compared with a younger group with a mean age of approximately 25 years. The age difference between the two groups is rather large. The aging effect on the two visual pathways in the intermediate age range remains unclear. In the current study, one aim was to examine the age-related changes in contrast sensitivity in the MC and PC visual pathways in a more continuous age range, and to examine whether contrast sensitivity in these visual pathways manifest a gradual age-related change or a significant, abrupt change in a certain age range. Considering that contrast sensitivity reduction at the overall perceptual level has been reported to start at 40 to 50 years of age [4, 18, 19], reduction of contrast sensitivity in the two visual pathways may start to occur at a similar age.

In addition, recent studies by our group showed that flicker adaptation reduced contrast sensitivity in the MC pathway but not in the PC pathway [30, 31]. Flicker adaptation is an exposure to high temporal contrast. The desensitization effect from flicker adaptation is thought to result from suppression of contrast responses of the ganglion cells and LGN cells in the MC pathway after adapting to high temporal contrast [22]. Flicker is a useful tool to study visual processing related to temporal vision. Studies have reported that patients with certain ocular diseases (e.g., ocular hypertension, glaucoma, and AMD) were less sensitive to flicker [32, 33], and flicker could be used in clinical tools to assist diagnosis or treat eye disease [34]. For instance, flicker glasses are currently available as a treatment method for amblyopia [35, 36]. Thus, flicker could be a useful tool scientifically and clinically. However, it is not well understood how exposure to flicker may affect visual functions at different ages. A few earlier studies showed that older adults processed flicker differently than younger adults, with older adults showing decreased sensitivity to flicker [37–40]. Older adults may also adapt to flicker differently than younger adults. It is important to better understand the age-related effect of flicker adaptation on visual functions so that appropriate consideration could be given if flicker is used as a clinical tool for people at different ages. Thus, a second aim of the current study was to use the same pedestal paradigms to investigate whether exposure to flicker affects contrast processing in visual pathways differently at different ages. As it was reported in earlier studies that older adults were less sensitive to flicker [37, 38], it was hypothesized that flicker adaptation affects contrast processing less in older adults than in younger adults. Therefore, the desensitization effect from flicker adaptation may be reduced in older adults.

## Methods

### Observers

Forty-five observers (age 39 ± 15 years) were recruited from employees, students, and patients at the Illinois College of Optometry. There were 16, 11, 7, and 11 observers in the age groups of 20–30 years, 31–40 years, 41–50 years, and >50 years (53–84 years), respectively. All observers had normal or corrected-to-normal visual acuity and were free of ophthalmic conditions. Written informed consent was obtained from all observers and they received monetary compensation for their participation in the study. The study protocol was approved by the Institutional Review Board of the Illinois College of Optometry and was in compliance with the Declaration of Helsinki.

### Apparatus

The visual stimuli were presented on an NEC 21” MultiSync FE771 (Tokyo, Japan) CRT color monitor (refresh rate 60 Hz) controlled by an iMac computer. CRT calibrations included the spectral outputs of the red, green, and blue guns of the CRT measured with a Photo Research PR-670 Spectrophotometer, and the linearity of the light outputs of each gun measured at 1,024 light levels using an International Light Radiometer/Photometer (IL-1700). A gaming console control (Logitech PRECISION) was connected to the computer to input observers’ responses.

### Visual stimuli and procedure

**Fig 1:**
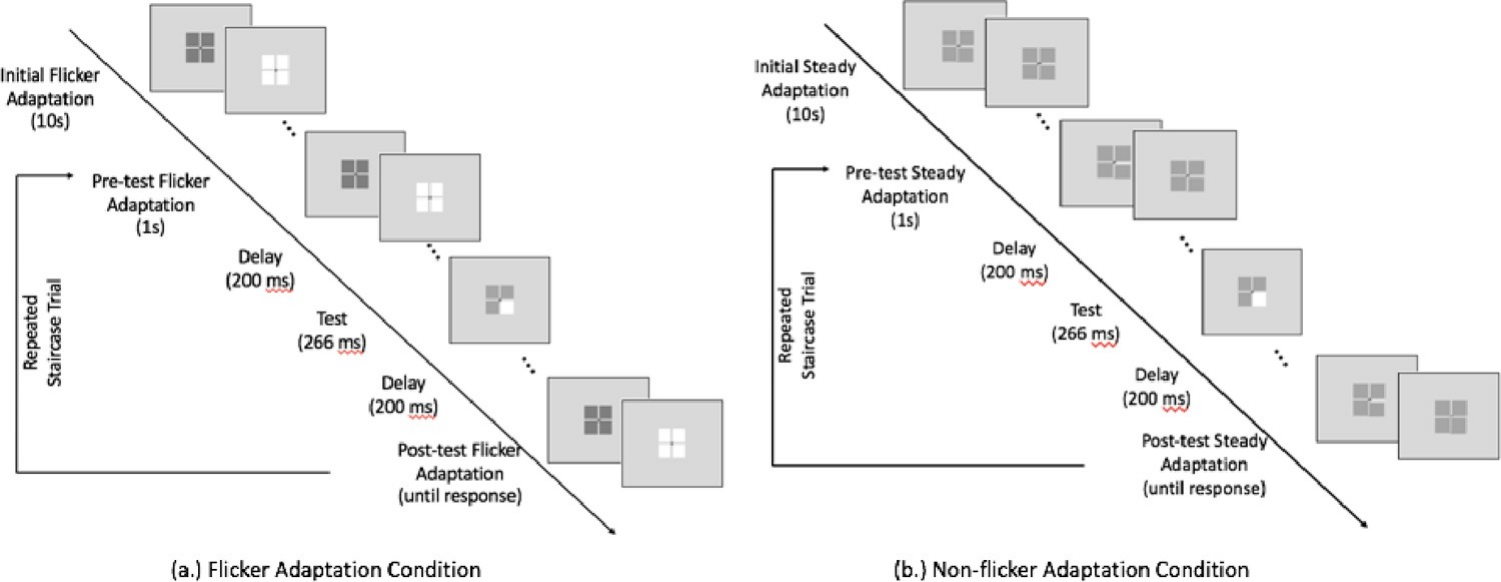
Schematic diagrams of the testing conditions in the steady- and pulsed-pedestal paradigms: (a) the flicker adapting condition and (b) the no-flicker condition.

Contrast discrimination thresholds in the MC and PC pathways were measured using two slightly different psychophysical contrast discrimination paradigms [41]: 1) the steady-pedestal paradigm to measure thresholds in the MC pathway; and 2) the pulsed-pedestal paradigm to measure thresholds in the PC pathway. A schematic diagram of the paradigms is shown in Fig 1. The visual stimulus consisted of a pedestal array of four equal-sized squares each subtending 1^°^ × 1^°^ in visual angle, surrounded by a homogeneous achromatic rectangular field of 15.0 cd/m^2^. There was a 0.089^°^ separation between squares, and a fixation cross of 0.089^°^ × 0.089^°^ was presented at the center of the array throughout the experiment.

In both the steady-pedestal and pulsed-pedestal paradigms, a measurement session started with a 10-second initial adaptation phase, in which either a 7.5 Hz pedestal flicker in the flicker adaptation condition (Fig 1A) or a steady-luminance pedestal in the non-flicker adaptation condition (Fig 1B) was presented. The initial adaptation was then followed by a 2-yes-1-no double-randomized staircase procedure that allowed estimation of the contrast discrimination threshold from a predefined pedestal luminance. In each staircase trial, a pre-test adaptation that was the same as the initial adaptation but shorter (1s) preceded the pedestal test stimulus array which was presented briefly for 266 ms. In the stimulus array, three of the four squares had a predefined pedestal luminance and one square (i.e., the test square) had an incremental luminance difference from the other three. The predefined pedestal luminance could be 15.0 (only tested in the steady-pedestal paradigm), 16.86, 18.88, 21.19, or 23.77 cd/m^2^. After the pedestal test stimulus, a post-test adaptation that was the same as the initial adaptation was presented until the observer responded. Observers were instructed to identify the test square in a four-alternative-forced-choice (4AFC) task by pressing one of the four buttons on a gamepad. In other words, the observer’s task was to report which square differed in brightness from the remaining squares. The luminance difference between the test square and the other three squares varied from trial to trial and was determined by the staircase procedure. The duration of the staircase procedure depended on the individual. On average, it took approximately 70 trials for the staircase procedure to converge for each luminance level, and 280 to 350 trials in total for one session (i.e., one adaptation condition with four or five predefined pedestal luminance levels, for the pulsed- or steady-pedestal paradigm, respectively).

The steady- versus pulsed-pedestal paradigms differed in the adaptation phase. In the flicker adaptation condition, the steady-pedestal paradigm had a flicker with a time-averaging luminance that was the same as a predefined pedestal luminance; by contrast, the pulsed-pedestal paradigm had a flicker with a time-averaging luminance that was the same as the background luminance. In the non-flicker adaptation condition, the pedestal had a luminance that was the same as a predefined pedestal luminance during adaptation in the steady-pedestal paradigm, but had a luminance that was the same as the background luminance in the pulsed-pedestal paradigm (see S1 and S2 Videos).

Each observer participated in the study on three separate days. On each day, four randomized-order sessions were conducted for the two pathways (MC vs. PC) and two adaptation conditions (non-flicker vs. flicker). In each session, the four or five predefined pedestal luminance levels were presented in different blocks, and the order of the luminance blocks were randomized from session to session. Therefore, contrast discrimination thresholds for each pedestal luminance level for each adaptation condition (flicker or no-flicker) in each paradigm (steady- or pulsed-pedestal) were measured three times for each observer, and an average of the three values was then used as the final threshold estimate for that pedestal luminance level. The total duration of the study was about 5–6 hours for each observer, with about 1.5–2 hours per day including practice. Other details of the flicker adaptation and pedestal paradigms are the same as published in our previous studies (see Zhuang et al., 2012*;* Pokorny and Smith, 1997; for the detailed rationale and theory behind the paradigms).

### Data analysis

Contrast discrimination thresholds from the two paradigms were analyzed separately for each observer. Thresholds for each pedestal luminance level in each adaptation condition for each paradigm were estimated and were fitted by two primate physiology-based models (see Pokorny and Smith, 1997; Pokorny, 2011; for detailed rationale and theories underlying these models): Eq 1 for the steady-pedestal paradigm, and Eq 2 for the pulsed-pedestal paradigm.

$$\log(\Delta I)=K_{s}+\log(I)$$ Eq 1

$$\log(\Delta I)=K_{p}+\log[{\left(C+C_{sat}\right)}^{2}]‐\log(C_{sat})$$ Eq 2

where *ΔI* represents the threshold estimate at the pedestal luminance level (*I*), C represents the Weber contrast between the predefined pedestal luminance and the background luminance, the free parameters -*K*_*s*_, -*K*_*p*_, and -*log*(*C*_*sat*_) are linearly related to contrast sensitivity in the MC pathway, contrast sensitivity in the PC pathway, and contrast gain in the PC pathway, respectively ^42^. A least-squares method was used for fitting each observer’s MC and PC data separately. After model fitting, the free parameters -*K*_*s*_, -*K*_*p*_, and -*log*(*C*_*sat*_) were then subjected to statistical testing for examination of the age and flicker adaptation effects. We applied the method of Linear Mixed Model (LMM) with maximum likelihood to analyze each of the three parameters (-*K*_*s*_, *-K*_*p*_, and -*log*(*C*_*sat*_)) with Age, Flicker Adaptation, and Age × Flicker Adaptation interaction as the fixed effects and observers as a random effect to account for the observer effect on each parameter. Age was entered as a continuous variable in the main LMM model, and as four different age groups in an additional LMM analysis. An alpha level of .05 was selected for all analyses. SPSS Statistics 25.0 (IBM Corp., Armonk, NY, USA) was used.

## Results

**Fig 2:**
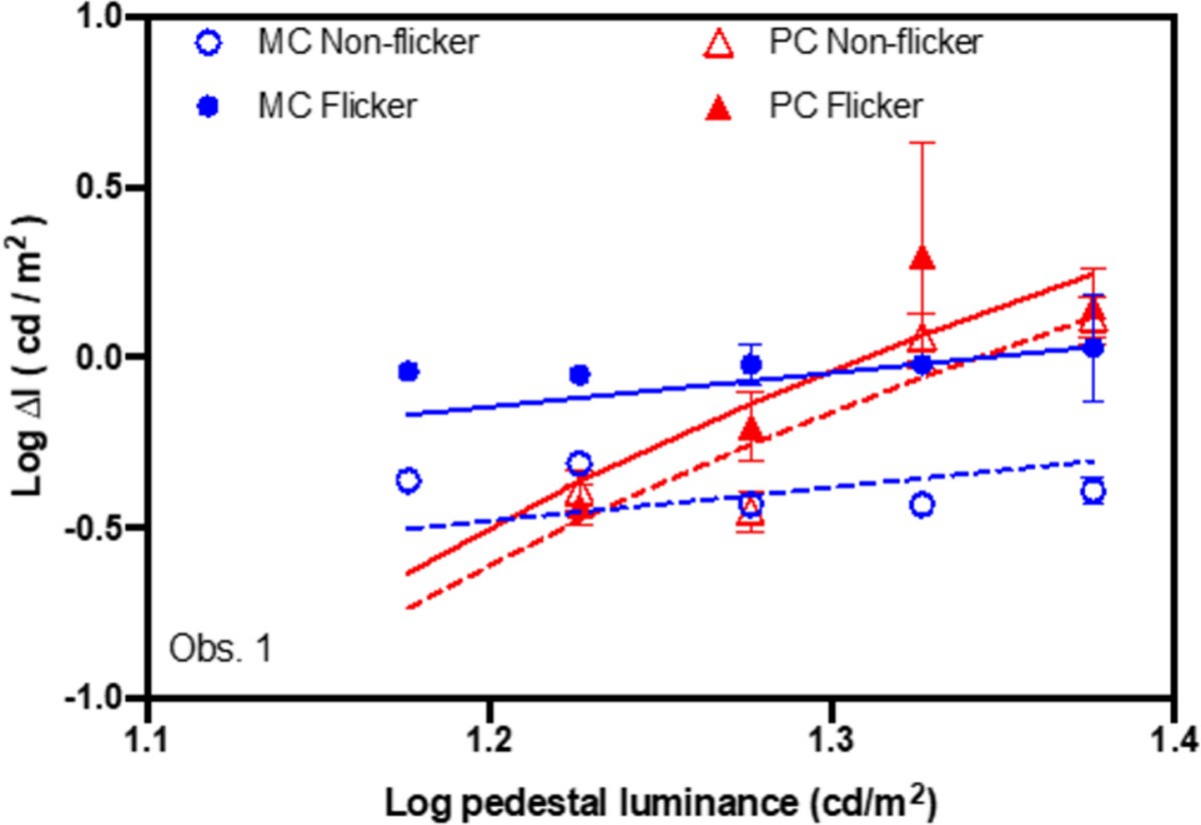
An observer’s steady- and pulsed-pedestal threshold data and model fits: (1) steady-pedestal paradigm (MC pathway) non-flicker adaptation condition (open circle); (2) steady-pedestal paradigm (MC pathway) flicker adaptation condition (filled circle); (3) pulsed-pedestal paradigm (PC pathway) non-flicker adaptation condition (open triangle); (4) pulsed-pedestal paradigm (PC pathway) flicker adaptation condition (filled triangle). Lines are the model fits from Eqs (1) and (2).

All individual observers’ threshold data showed the typical patterns from the steady- and pulsed- pedestal paradigms [41, 42]. An observer’s data is shown in Fig 2 as an example, and all observers’ data is shown in the S1 Appendix. The log contrast discrimination thresholds are plotted as a function of log pedestal luminance, and the model fits from Eq 1 for the steady-pedestal data and from Eq 2 for the pulsed-pedestal paradigms are also shown in Fig 2. The threshold data from the steady-pedestal paradigm could be described by a straight line with a slope of 1 (Eq 1), following Weber’s law. The thresholds from the pulsed-pedestal paradigm also increased with increasing pedestal contrast but had a steeper slope and formed a curve (Eq 2). Each observer’s MC contrast sensitivity (-*K*_*s*_), PC contrast sensitivity (-*K*_*p*_), and PC contrast gain (-*log*(*C*_*sat*_)) parameters were estimated using Eqs 1 and 2. These estimates were then analyzed using the LMM to examine the effects of age and flicker adaptation. Descriptive statistics of the parameters (-*K*_*s*_, -*K*_*p*_, and -*log*(*C*_*sat*_)) are presented in Table 1.

**Table 1:** Descriptive statistics for the free parameters.

	Non-flicker adaptation condition		Flicker adaptation condition	
	Mean	SD	Mean	SD
***-K*_*s*_**	1.48	0.17	1.03	0.17
***-K*_*p*_**	0.84	0.44	0.72	0.60
***-log(C*_*sat*_ *)***	0.34	0.52	0.15	0.49

### MC pathway

**Fig 3:**
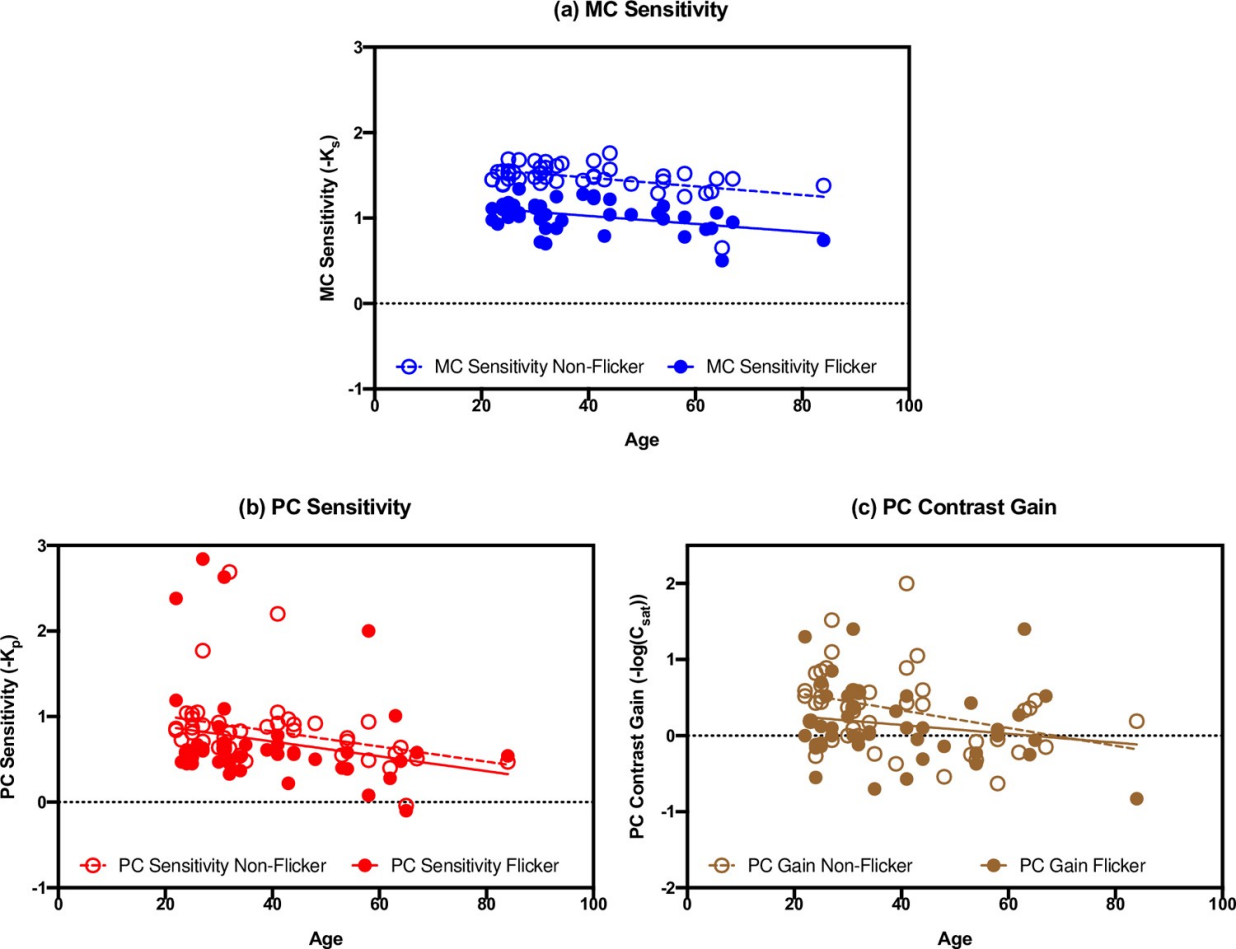
The best fitting parameters for all observers with the effects of age and flicker adaptation: (a) parameter data for MC contrast sensitivity (-*K*_s_), (b) parameter data for PC contrast sensitivity (-*K*_*p*_), (c) parameter data for PC contrast gain (-*log*(*C*_*sat*_)). Open circles represent the data from the non-flicker adaptation condition, while filled circles represent the data from the flicker adaptation condition.

LMM analysis showed that the main effect of Age was significant [*F(1*,*45) = 13*.*946*, *p = 0*.*001*]. Contrast sensitivity decreased as age increased. The main effect of Flicker Adaptation was also significant [*F(1*,*45) = 51*.*891*, *p < 0*.*001*], indicating significantly lower contrast sensitivity in the flicker condition than in the non-flicker condition (Fig 3A). However, the interaction effect between Flicker Adaptation and Age was not significant [*F(1*,*45) = 0*.*058*, *p = 0*.*811*], suggesting a similar magnitude of flicker adaptation effect at different ages.

### PC pathway

LMM analysis on PC sensitivity showed that the main effect of Age was significant [*F(1*,*45) = 5*.*380*, *p = 0*.*025*]. PC contrast sensitivity decreased as age increased. However, the main effect of Flicker Adaptation was not significant [*F(1*,*45) = 0*.*217*, *p = 0*.*644*], showing no significant difference between PC sensitivity under the flicker condition versus the non-flicker condition (Fig 3B). The interaction effect between Flicker Adaptation and Age also was not significant [*F(1*,*45) = 0*.*001*, *p = 0*.*977*]. That is, a significant impact from flicker adaptation on PC contrast sensitivity was not observed for any age. Similar results were found on PC contrast gain. The main effect of Age [*F(1*,*45) = 6*.*219*, *p = 0*.*016*] was significant, but neither the main effect of Flicker Adaptation [*F(1*,*45) = 2*.*54*, *p = 0*.*118*], nor the interaction effect [*F(1*,*45) = 0*.*830*, *p = 0*.*367*, Fig 3C] was significant. That is, PC contrast gain decreased as age increased, but flicker adaptation did not significantly affect the PC contrast gain at any age.

**Fig 4:**
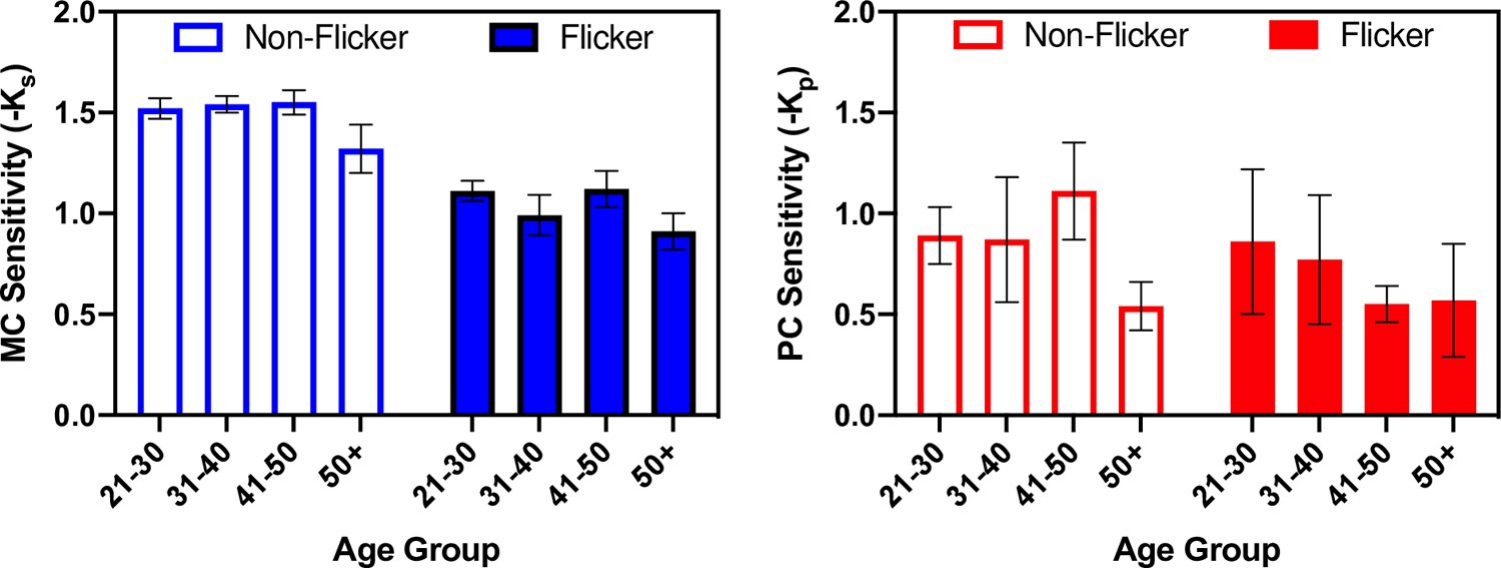
Averaged contrast sensitivity parameter data (Mean and SD) for the MC and PC pathways by age group.

An additional set of LMM analyses was conducted by separating the observers into four different age groups (Fig 4): 20–30 years, 31–40 years, 41–50 years, and >50 years, to evaluate the effects of Age Group, Flicker Adaptation, and the Age Group × Flicker Adaptation interaction. The analyses showed significant main effects of Flicker Adaptation [*F(1*,*45) = 39*.*741*, *p <0*.*001*], and Age Group [*F(3*,*45) = 6*.*459*, *p = 0*.*001*] for the MC pathway. Bonferroni post hoc comparison showed that contrast sensitivity for the >50 age group was significantly lower than that of the three younger groups, whereas the three younger groups did not significantly differ from each other. On the other hand, for PC contrast sensitivity and PC contrast gain, no significant main effects of Age Group or Flicker Adaptation were found [PC contrast sensitivity: *F(1*,*45) = 3*.*009*, *p = 0*.*090* for Flicker Adaptation, and *F(3*,*45) = 1*.*678*, *p = 0*.*185* for Age Group; PC contrast gain: *F(1*,*45) = 2*.*154*, *p = 0*.*149* for Flicker Adaptation, and *F(3*,*45) = 2*.*508*, *p = 0*.*071* for Age Group]. The interaction between Flicker Adaptation and Age Group was not significant for either the MC pathway [*F(3*,*45) = 2*.*265*, *p = 0*.*094*] or PC pathway [*F(3*,*45) = 1*.*472*, *p = 0*.*235* for contrast sensitivity, and *F(3*,*45) = 0*.*327*, *p = 0*.*806* for contrast gain].

### MC and PC contrast sensitivity comparison

LMM analysis was also applied to compare the contrast sensitivity between the MC and PC pathways. The difference in contrast sensitivity between the two pathways was significant in the non-flicker adaptation condition [*F(1*,*45) = 9*.*181*, *p = 0*.*004*] with higher contrast sensitivity in the MC pathway than in the PC pathway, but not in the flicker adaptation condition [*F(1*,*45) = 0*.*452*, *p = 0*.*505*]. The interaction effect between Age and Pathway was not significant in either adaptation condition [non-flicker: *F(1*,*45) = 0*.*946*, *p = 0*.*336;* flicker: *F(1*,*45) = 0*.*506*, *p = 0*.*481*].

## Discussion

An aging effect on the overall-level contrast sensitivity has been reported in many studies [4, 15–17]. In particular, aging has shown an obvious effect on high spatial frequencies. It was proposed that depending on the spatial frequency, different mechanism(s) may be involved in the process. An increase in internal noise may play a significant role in the loss of contrast sensitivity for high spatial frequencies, whereas low calculation efficiency may contribute to the decline of contrast sensitivity for relatively low spatial frequencies [43–46]. It was also suggested that optical factors play major roles in age-related contrast sensitivity deterioration [4, 15, 19]; however, other studies demonstrated the contribution of neural factors [20, 21].

In the present study, we aimed to investigate the aging effects specific to the MC and PC visual pathways, as well as the impact of a rapid temporal flicker adaptation on contrast sensitivity in the two pathways at different ages. The pedestal paradigms to measure contrast sensitivity separately in the two visual pathways [41] were used for a continuous age range of observers. The results showed significant age-related changes of contrast sensitivity in both the inferred MC and PC visual pathways. As shown in Fig 3, under both non-flicker and flicker adaptation conditions, contrast sensitivity in the MC and PC pathways decreased as age increased. However, when separating the observers into different age groups, a significant effect of age group was observed in the MC pathway but not the PC pathway (Fig 4). The PC contrast sensitivity data showed large variation among individuals mainly due to a few participants who had high PC sensitivity. We conducted an additional LMM analysis on the PC data after removing the individuals whose PC sensitivity was outside 3 standard deviations, and the results remained unchanged. Together, these results may suggest that contrast sensitivity in both MC and PC pathways decreases as age increases in general, and there may be a more dramatic reduction of contrast sensitivity in the MC pathways for those >50 years old. Age-related contrast sensitivity reduction observed at the overall perceptual level likely results from contrast sensitivity reduction in both the MC and PC visual pathways.

Anatomical and physiological studies have reported neuronal changes in visual pathways due to aging. For instance, age-related reduction of retinal ganglion cell axon density in humans has been reported [47]. Studies in other primates also reported age-related decrease of neuron density in both the magnocellular and parvocellular layers of the lateral geniculate nucleus (LGN) [48]. Benedek et al. (2016) [49] showed that neurons in the two visual pathways started to show signs of aging beyond 50 years of age, in particular in the MC pathway. Consistent with these studies, the present study adds to the literature from a human psychophysics functional perspective on how contrast sensitivity in visual pathways changes in a continuous age range.

The other goal of the current study was to assess whether rapid temporal flicker adaptation interacts with the aging process on contrast sensitivity in the two visual pathways. Our results showed that flicker adaptation had a similar desensitizing effect on the MC pathway for all ages, but no effect on the PC pathway for any age. In our study, the spatial frequency of the pedestal was relatively low (e.g., ~0.5 cpd). Other studies have shown that the effect of aging on low spatial frequencies depended on the specific temporal modulation condition, and it is more prominent under rapid temporal modulation, which requires both spatial contrast sensitivity as well as temporal sensitivity [50–52]. Earlier studies showed that older adults processed temporal information differently than younger adults. Older adults showed decreased temporal sensitivity and were less sensitive to flicker [37, 38]. In the present study, another relevant process is visual adaptation. Visual adaptation is a natural process allowing the visual system to maintain its sensitivity to changes in the environment. Some recent studies have demonstrated that aging did not affect the visual adaptation process. For instance, Lek et al. (2014) [53] demonstrated no difference in contrast adaptation for presentation of grating stimuli in healthy older adults as compared to younger adults. In our study, our observers adapted to a temporal flicker. Our results showed that flicker adaptation seemed to have a similar impact on contrast sensitivity at different ages. This finding may indirectly suggest that aging does not affect the process of visual adaptation to a rapid temporal flicker. The flicker used here was 7.5 Hz and 50%, the stimulus size was 1° × 1°, and the stimulus duration was 266 ms. These specific stimulus characteristics may be important factors that could impact the specific adaptation status of the visual system at different ages. The effect of flicker adaptation is thought to be due to suppression of contrast responses in ganglion cells and LGN cells [22]. It is known that cells in the MC and PC pathways show different response characteristics to stimuli of different temporal frequencies. Cells in the MC pathway respond better to high temporal frequencies, whereas cells in the PC pathway respond better to low temporal frequencies [54]. Further study will be needed to continue exploring the age-related effects of flicker adaptation on different temporal frequencies.

A limitation of the present study is that only one stimulus size (a pedestal square of 1° × 1°) was tested, which provided findings for a low spatial frequency (~0.5 cpd). It is known that cells in the MC and PC pathways have distinct spatial properties. Cells in the PC pathway show higher spatial resolution than cells in the MC pathway [55, 56]. The age-related effects on the two visual pathways may depend on spatial frequency. Previous studies have shown that age-related contrast sensitivity deterioration at the overall perceptual level starts at high spatial frequencies and then progresses to all spatial frequencies at a later age [4, 18, 19]. This pattern of age-related effects may be the same in the two pathways. Further investigation will be needed using more spatial frequencies to evaluate age-related effects on the entire contrast sensitivity function in the two visual pathways. Another limitation of the present study was the relatively small sample size of the 40–50 and >50 age groups due to greater difficulty recruiting in these age groups. A more comprehensive study with more experimental conditions and larger sample sizes is warranted in the future.

## Supporting information

S1 Video Pulsed-pedestal task video demonstrates a trial in the pulsed-pedestal paradigm. (MOV)

S2 Video Steady-pedestal task video demonstrates a trial in the steady-pedestal paradigm. (MOV)

S1 Data (XLSX)

S1 Appendix All observers’ data by age group. (DOCX)
