# Supplementary material for: Aging effects on contrast sensitivity in visual pathways: A pilot study on flicker adaptation
Source: PLoS One. 2021 Dec 31;16(12):e0261927. doi: 10.1371/journal.pone.0261927 (PMC8719693; doi:10.1371/journal.pone.0261927)
Supplement: S1 Appendix — (DOCX) [file pone.0261927.s004.docx]

**S4 Appendix. All observers’ data by age group.**

(a) Age group: 20-30 years

(b) Age group: 30-40 years

(c) Age group: 40-50 years

(d) Age group: >50 years

Appendix Figure. All observers’ steady- and pulsed-pedestal threshold data and model fits by age group: (a) age group 20-30 years, (b) age group 30-40 years, (c) age group 40-50 years, (d) age group >50 years. Open circles represent data from the steady-pedestal paradigm (MC pathway) non-flicker adaptation condition; filled circles represent data from the steady-pedestal paradigm (MC pathway) flicker adaptation condition; open triangles represent data from the pulsed-pedestal paradigm (PC pathway) non-flicker adaptation condition; filled triangles represent data from the pulsed-pedestal paradigm (PC pathway) flicker adaptation condition. Data point circled in yellow was considered an outlier and excluded from analysis. Lines are the model fits from Eq. (1) and (2). Three quartiles summary for the R^2^ of the model fits for all individuals: median R^2^ = 0.86, IQR = (0.62, 0.96) for the MC models; median R^2^ = 0.89, IQR = (0.72, 0.96) for the PC models.
